# Genomic Profiling of Adults with Pharmacoresistant Genetic Generalized Epilepsy

**DOI:** 10.3390/brainsci16050521

**Published:** 2026-05-14

**Authors:** Benjamin L. Kidder, Jian Xu, Rui Geng, Hunter Dlugas, Anusha Vavilikolanu, Wei Chen, Vibhangini S. Wasade

**Affiliations:** 1 Department of Oncology, Wayne State University School of Medicine, Detroit, MI 48201, USA; ruigeng@wayne.edu (R.G.); avavili@wayne.edu (A.V.); weichen@wayne.edu (W.C.); 2 Karmanos Cancer Institute, Wayne State University School of Medicine, Detroit, MI 48201, USA; fy7392@wayne.edu; 3 Henry Ford Comprehensive Epilepsy Program, Department of Neurology, Henry Ford Hospital, Detroit, MI 48202, USA; jxu3@hfhs.org; 4 Department of Neurology, Wayne State University School of Medicine, Detroit, MI 48201, USA; 5 Department of Neurology, Michigan State University, Lansing, MI 48824, USA

**Keywords:** genetic generalized epilepsy, pharmacoresistant epilepsy, whole-genome sequencing, coding variants, pharmacogenomics

## Abstract

**Highlights:**

**What are the main findings?**

• Whole-genome sequencing in adults with pharmacoresistant GGE identified 133 recurrent deleterious coding variants across 69 genes shared by most patients
• Prioritized variants converge on pathways involving metabolism and drug absorption, neuroimmune signaling, and ion transport

**What are the implications of the main findings?**

• Findings support a multifactorial, pathway-level basis of pharmacoresistance rather than single-gene effects
• Results highlight the potential of whole-genome sequencing to inform precision medicine approaches and guide future validation studies

**Abstract:**

**Background/Objectives**: Genetic generalized epilepsies (GGE) often remit in childhood, yet a subset of adults remain pharmacoresistant with substantial morbidity. The genetic basis of adult pharmacoresistant GGE is poorly defined. This descriptive study used whole-genome sequencing (WGS) to identify recurrent coding variants and pathways associated with pharmacoresistant adult GGE. **Methods**: WGS was performed in ten racially diverse adults (mean age 37.2 years; range 20–52) with electroencephalographically confirmed, pharmacoresistant GGE (mean onset 13.7 years). Analysis prioritized variants present in at least 80% of participants and which were either (i) missense variants predicted deleterious with ANNOVAR or (ii) loss-of-function variants predicted high-impact from snpEff. Pathway enrichment and overlap with a commercial clinical epilepsy gene panel were assessed. **Results**: Filtering identified 133 unique, deleterious coding variants across 69 genes shared by at least eight participants. Four genes (APOL4, KMT2C, SON, VDR) overlapped a clinical epilepsy panel, supporting the capacity of WGS to recover clinically relevant loci. Prioritized loci implicated gastrointestinal and metabolic regulators (e.g., MUC6, PNLIPRP2), chemosensory receptors (OR10D3, OR8U1, TAS2R19), neuroimmune mediators (LILRA2, SIGLEC12, OAS2), and ion transporters (KCNJ12, P2RX5, RHBG), consistent with multifactorial mechanisms of pharmacoresistance. **Conclusions**: This exploratory WGS study focused exclusively on adults with pharmacoresistant GGE, revealing shared high-impact variants and convergent pathways spanning absorption/metabolism, vitamin D signaling, immunity, and ion transport. Findings broaden the genetic landscape of pharmacoresistant GGE while motivating validation in larger, multiethnic cohorts.

## 1. Introduction

Epilepsy, a chronic neurological disorder affecting over 3.4 million people in the United States (including 3 million adults), imposes significant morbidity and mortality, with one-third of cases progressing to pharmacoresistant epilepsy, usually described as intractable epilepsy, defined by persistent seizures despite trials of ≥2 appropriately selected anti-seizure medications (ASMs) [1,2]. Adults with drug-resistant epilepsy face devastating consequences, including heightened psychosocial disability, reduced quality of life, and a 15-fold increased risk of sudden unexpected death in epilepsy (SUDEP) [3,4]. Genetic generalized epilepsy (GGE), historically termed idiopathic generalized epilepsy (IGE), represents a distinct subset characterized by generalized seizure types (e.g., absence, myoclonic, tonic–clonic) and 2.5–5.5 Hz spike-wave discharges on electroencephalography [5]. While GGE typically manifests in childhood and remits by adulthood, 10–30% of cases persist into adulthood or emerge de novo in later life, often exhibiting resistance to ASMs [6,7], suggesting a possible underlying genetic basis for intractability as well.

GGE is a group of complex polygenic disorders [8]. A Genome-Wide Association Study (GWAS) meta-analysis of international cohorts using single-nucleotide polymorphism (SNP) arrays identified common risk variants for GGE, most prominently implicating the VRK2/FANCL locus at 2p16.1 [9]. Another study that applied GWAS mega-analysis with imputed SNPs and gene prioritization strategies revealed 11 genome-wide significant loci, seven of which were novel [10]. Despite these advances, genetic predictors of pharmacoresistance in GGE remain poorly characterized. Recent studies have implicated variants in epigenetic modifier genes, immunological pathways, and drug metabolism as possibly contributing to the emergence of pharmacoresistant epilepsy [8]. Identification of such variants in GGE cohorts may enable earlier targeted therapeutic strategies and help mitigate chronic, uncontrolled seizures.

In this pilot study, we conducted whole-genome sequencing (WGS) in 10 adults with pharmacoresistant GGE, with the objective of identifying common deleterious variants and assessing their potential roles in drug resistance. The results of this investigation provide a foundation for future collaborative, large-scale studies aimed at developing precision medicine strategies and improving long-term outcomes in adults with drug-resistant GGE.

## 2. Materials and Methods

### 2.1. Patient Population and Data Collection

We utilized a convenience sampling approach for this pilot study. Participants were identified retrospectively through systematic chart review of adult patients seen at Henry Ford Epilepsy clinics between November 2022 and July 2023. A total of 18 patients met eligibility criteria and were invited to participate. Of these, 10 patients (55.6%) provided informed consent and completed the blood draw on the same day. The remaining eight patients either declined participation or did not respond to the invitation. All ten collected specimens successfully underwent laboratory processing and met quality-control thresholds for whole-genome sequencing (WGS), resulting in a final analytical sample of *n* = 10. No enrollment caps, quotas, or selection targets were applied based on sex, race, ethnicity, or ancestry.

Inclusion criteria required each patient to have persistent seizures despite trials of two or more anti-seizure drugs (ASMs). Exclusion criteria included focal epilepsy, well-controlled generalized epilepsy, or inability to travel for a single blood draw. No comorbidities or prior genetic testing results were used as exclusion criteria.

Eligible individuals were contacted, provided informed consent, and visited the Henry Ford Neurology Clinic for a one-time blood draw. Demographic and clinical information was extracted from electronic medical records, including gender, age at seizure onset, seizure frequency, and previous ASM trials. This study was approved by the Henry Ford Internal Funding Grant and was regulated by the Office for Human Research Protections (OHRP). All methods were performed in compliance with institutional guidelines and were approved by Henry Ford Health System IRB (Protocol #15939). Informed consent was obtained from all subjects involved in the study. Participants were compensated for their time and travel.

Pharmacoresistance was defined according to International League Against Epilepsy criteria as failure of at least two appropriately chosen and tolerated antiseizure medications. For all participants, treatment histories were reviewed to confirm failure of syndrome-appropriate ASMs commonly used for genetic generalized epilepsy (e.g., valproate, levetiracetam, lamotrigine, topiramate), either as monotherapy or in combination.

### 2.2. Sample Collection and DNA Extraction

Peripheral blood was collected using BD Vacutainer CPT Mononuclear Cell Preparation Tubes (Becton, Dickinson and Company, Franklin Lakes, NJ, USA). Within two hours of collection, tubes were centrifuged for 20–30 min at 1500–1800 RCF. Peripheral blood mononuclear cells (PBMCs) were resuspended in plasma by gentle inversion. Genomic DNA was extracted from PBMCs using the QIAamp DNA Blood Mini Kit (Qiagen, Hilden, Germany). DNA concentration was quantified using a Qubit dsDNA High Sensitivity assay (Thermo Fisher Scientific, Waltham, MA, USA). Samples met standard quality thresholds for sequencing, including adequate concentration and minimal degradation, and were stored at −80 °C prior to library preparation.

### 2.3. Whole-Genome Sequencing

Genomic DNA was randomly sheared into short fragments (~350 bp). The fragmented DNA underwent end repair and 3′ A-tailing, then was ligated to Illumina sequencing adapters. Adapter-ligated fragments were PCR-amplified, size-selected and purified using magnetic bead cleanup. Library concentration was quantified by Qubit dsDNA HS assay and real-time PCR, and fragment size distribution was assessed on an Agilent Bioanalyzer (Agilent Technologies, Santa Clara, CA, USA). Final libraries were subjected to paired-end sequencing on an Illumina NovaSeq X Plus platform (Novogene, Beijing, China) according to the manufacturer’s protocols. The nf-core/sarek pipeline (v3.2.3) was used to process raw sequencing data [11,12]. This involved aligning reads to the GRCh38 reference genome using the Burrows-Wheeler Aligner [13] (bwa mem), marking duplicate reads (gatk MarkDuplicates), sorting sam files by coordinate (gatk SortSam), and recalibrating base quality scores (gatk BaseRecalibrator + gatk ApplyBQSR) according to Genome Analysis Toolkit (GATK) Best Practices [14].

### 2.4. Variant Calling and Annotation

Germline variants were identified with GATK [15,16] HaplotypeCaller (v4.4.0.0) within the nf-core/sarek pipeline (v3.2.3) executed in Nextflow (v23.04.1). Resulting Variant Call Format (VCF) files for each sample were filtered to remove low-quality variants (quality score < 100, read depth < 20, or proportion of major alternate allele depth <1/6). Remaining variants underwent functional annotation using both ANNOVAR [17] (June 2020 version)- a popular tool which annotates each SNV with deleteriousness scores from various algorithms and provides variant frequency in 1000 Genomes (phase 3) and gnomAD v4.1—and snpEff [18], which predicts HIGH impact variants with loss-of-function such as frameshifts and stop gains. Hence, information such as predicted coding impact (for example, synonymous, missense, frameshift, etc.) and putative deleteriousness scores were generated.

### 2.5. Ancestry Estimation

A biogeographical ancestry analysis (i.e., admixture analysis) was performed using the R package EthSEQ [19] (v3.0.1) with the hg38 GENCODE exome reference. Exact binomial tests were used to compare the frequency of each ancestry group in our pharmacoresistant GGE cohort with the corresponding frequency in a given reference group. The three reference groups considered were from the 1000 Genomes Project [20], gnomAD [21], and GGE cases from Epi25 [22]. The Benjamini–Hochberg method was used to adjust *p*-values.

### 2.6. Defining Common Deleterious Variants

To identify variants of interest, we applied two concurrent decision rules. First, variants were retained only if they occurred in at least eight of the ten samples (≥80% frequency). This threshold implies that the lower bound of the 95% Wilson confidence interval for true variant prevalence in this pharmacoresistant population exceeds 49%, thereby enriching for highly prevalent variants unlikely to represent technical artifacts or rare background variants. Second, we required that these shared variants be predicted deleterious, defined either by high-impact consequences in snpEff (i.e., loss-of-function variants such as frameshift, nonsense, canonical splice-site) or by deleterious missense predictions using ensemble machine-learning classifiers (radial SVM and logistic regression) from ANNOVAR, selected for their strong performance in identifying deleterious nonsynonymous single nucleotide variants (SNVs) [23]. Thus, only variants meeting both frequency and deleteriousness criteria were advanced for downstream analysis. Variants were prioritized for research purposes based on predicted functional impact and recurrence criteria and were not formally classified under ACMG/AMP clinical pathogenicity guidelines.

### 2.7. Comparison with Reference Population Frequencies

For the identified common deleterious variants, allele frequencies were compared to those reported by (i) the 1000 Genomes Project [20], (ii) the Genome Aggregation Database (gnomAD) [24], and (iii) individuals with GGE (not necessarily pharmacoresistant GGE) in the Epi25 Collaborative [22]. Exact binomial tests were conducted to determine whether the observed frequencies in this pharmacoresistant epilepsy cohort significantly diverged from those in the reference cohorts. *p* values were corrected for multiple testing using the Benjamini–Hochberg method. Variants without reported reference frequencies were excluded from statistical testing.

### 2.8. Functional Pathway Analysis

Shared high-impact genes (*n* = 69) were subjected to two complementary workflows. First, unsupervised clustering of Gene Ontology and pathway terms was performed in Metascape [25] using default settings. In parallel, targeted pathway enrichment was carried out in Enrichr [26,27]. We used Enrichr’s clustergram function to visualize gene-pathway relationships as a hierarchical heatmap, with color intensity proportional to −log_10_ (*p*-value).

## 3. Results

### 3.1. Patient Characteristics

Ten adults (age range 20–52 years, mean 37.2 years) with clinically pharmacoresistant and electroencephalographically confirmed genetic (former-idiopathic) generalized epilepsy (GGE) were included in this study (Table 1). The mean age of epilepsy onset was 13.7 years (range 4–31 years). Seven individuals were female, four (40%) self-identified as White, three (30%) as Black, one (10%) as Hispanic, and two (20%) as Other. All individuals had undergone trials of multiple antiseizure medications (ASMs), with a median of three prior regimens (range 1–6) and were receiving a median of three ASMs at sampling (range 2–5). The predominant seizure semiology was generalized tonic–clonic with absence seizures in five patients (50%), followed by isolated tonic–clonic seizures in two (20%) and tonic–clonic plus myoclonic seizures in two (20%); one patient (10%) exhibited combined tonic–clonic, absence, and myoclonic events. Table 1 summarizes the demographics, clinical characteristics, and treatment histories of this cohort.

### 3.2. Variant Filtering and Characteristics

After initial variant calling, an average of 5.21 × 10^6^ variants per sample (range 4.90 × 10^6^–5.98 × 10^6^) were identified. Subsequent prioritization identified 133 high-confidence deleterious variants shared across ≥80% of participants (Table S4). Following sequential quality and allele-frequency filtering steps, the total number of variants reduced to an average of 4.02 × 10^6^ variants across 10 samples (range 3.38 × 10^6^–5.13 × 10^6^) (Table S2).

Assessment of variant sharing across the ten patients revealed 11.40 × 10^6^ unique variant sites. Of these, 4.24 × 106 (37.2%) were limited to a single individual, while 409,811 (3.6%) were found in all ten patients. The number of shared variants decreased progressively from 1.56 × 10^6^ (13.7%) in two individuals to 527,994 (4.6%) in nine (Table S3). In particular, 584,927 (5.13%) variants were found in at least eight of the ten patients. Among variants retained after filtering, 133 high-confidence deleterious variants shared across ≥80% of participants were prioritized for downstream analysis (Table S4), involving 69 genes (Table S1).

Functional annotation of post-filtering variants revealed a predominance of noncoding changes (Table S5), as is expected for WGS data. On average, intergenic sites accounted for 2.23 × 10^6^ variants per sample (54.7%), and intronic sites for 1.43 × 10^6^ (34.9%). Noncoding RNA intronic variants averaged 262,118 per sample (6.4%), while 3′-UTR variants numbered 38,794 (0.95%) and 5′-UTR variants 6564 (0.16%). Upstream and downstream regulatory elements together comprised 53,545 variants (1.3%).

By contrast, exonic variants were rare, averaging 23,616 per sample, of which, on average, 11,655 were synonymous SNVs and 11,002 were nonsynonymous SNVs. Predicted loss-of-function events were infrequent (<0.01% each): splice-site altering variants averaged 121 per sample; frameshift deletions 117; frameshift insertions 82; stopgain 115; stoploss 17; and startloss 26 variants per sample.

Regarding exonic variations, a total of 68,911 were catalogued. The majority (53,826; 78.1%) were not SNVs with a deleterious prediction across nine in silico algorithms (sorting intolerant from tolerant (SIFT), Polyphen2_HumDiv (HDIV), Polyphen2_HumVar (HVAR), likelihood-ratio test (LRT), MutationTaster, MutationAssessor, functional analysis through hidden markov model (FATHMM), ensemble support vector machine with radial kernel, ensemble logistic regression), affecting 14,437 genes (580 known epilepsy genes). SNVs with one deleterious prediction comprised 7316 (10.6%) exonic variants across 4659 genes (105 epilepsy genes), while only 3167 (4.6%) exonic variants were SNVs with exactly two deleterious votes. High-confidence deleterious SNVs (five or more predictors) were markedly scarce, representing 721 (1.0%) at five predictors and dwindling to 39 (0.1%) at nine predictors, with few or no known epilepsy genes implicated as the prediction count rose (Table S6).

Concordant with in silico SNV deleteriousness estimation, snpEff-assigned HIGH-impact exonic variants averaged 278 per sample (range 230–320; 1.2%), moderate-impact variants averaged 10,811 (range 9829–12,370; 45.8%), low-impact variants 11,514 (range 10,417–13,312; 48.8%), and modifier-impact variants 997 (range 925–1143; 4.2%).

### 3.3. Admixture Analysis

**Figure 1:**
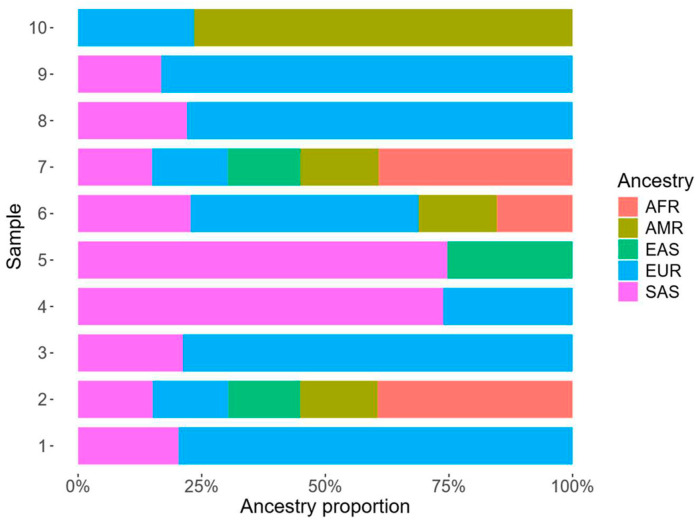
Ancestry estimates of pharmacoresistant GGE cohort estimated with EthSEQ. AFR: African; AMR: Admixed American; EAS: East Asian; EUR: European; SAS: South Asian.

Biogeographical ancestry inference using EthSEQ (v3.0.1) [19] assigned individuals in the pharmacoresistant GGE cohort to four ancestry groups: African (AFR), Admixed American (AMR), Asian (i.e., East Asian (EAS) and South Asian (SAS)), and European (EUR). The distribution of estimated ancestries was 20% AFR, 10% AMR, 20% ASN (EAS + SAS), and 50% EUR (Figure 1; Tables S7 and S8).

The ancestry distribution in the pharmacoresistant GGE cohort was compared to three reference cohorts: 1000 Genomes (*n* = 2184), gnomAD (*n* = 807,162), and epilepsy cases from Epi25 (*n* = 28,955). Exact binomial tests found no significant differences between the frequency of any particular ancestry group in our pharmacoresistant GGE cohort and any of the three reference groups. Overall, the pharmacoresistant GGE cohort exhibited ancestral composition without statistically significant enrichment or depletion of any major ancestry group relative to the three reference groups, although these comparisons should be interpreted cautiously due to the modest pharmacoresistant GGE cohort size (*n* = 10).

### 3.4. Identification of Shared High-Impact Variants

Filtering with both snpEff and ANNOVAR criteria yielded 133 unique high-impact coding variants (Table S4) mapping to 69 genes (Table S1). The distribution of variant types among these 133 prioritized variants is summarized in Table S9. Among these, ACACA, COL12A1, VDR, and ZNF717 emerged frequently, implicating pathways related to fatty-acid biosynthesis, extracellular matrix integrity, vitamin D signaling, and transcriptional regulation in the etiology of pharmacoresistant GGE. Among the 69 shared high-impact genes, four (APOL4, KMT2C, SON, and VDR) also appear on the LabCorp clinical epilepsy panel (Table S10). These genes are more broadly associated with diverse neurodevelopmental or metabolic phenotypes, and their presence in our cohort should be interpreted as preliminary signals rather than evidence for immediate clinical translation. We suggest that these overlaps highlight the capacity of WGS to recover genes included in some clinical testing frameworks, but further evaluation in larger, independent epilepsy cohorts is needed before any consideration for inclusion in diagnostic panels. This integrative approach highlights a core set of high-confidence genetic targets for future functional validation and precision diagnostics in pharmacoresistant GGE.

### 3.5. Pathway Enrichment Analysis

**Figure 2:**
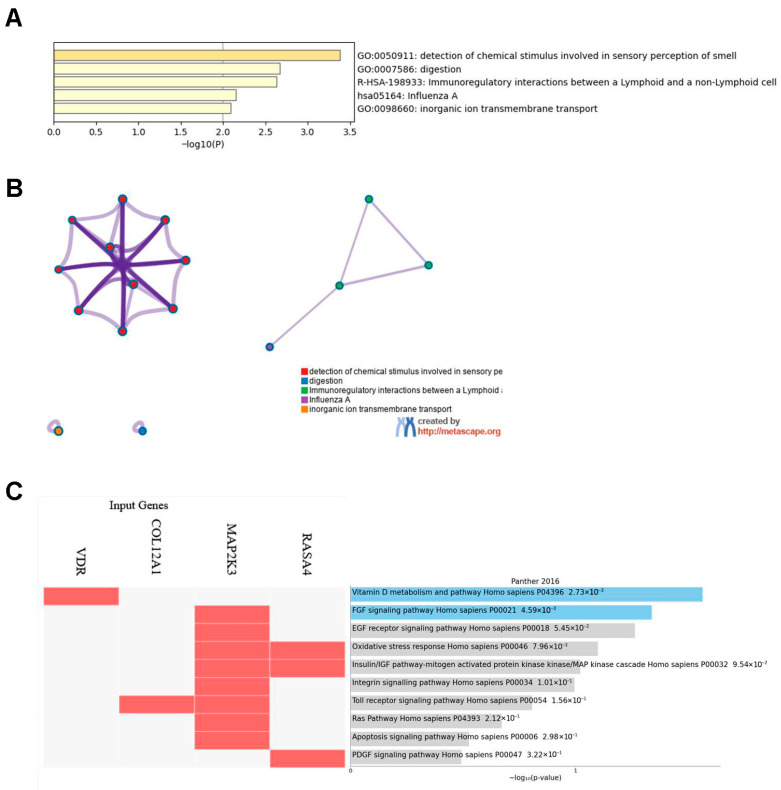
Enrichment analysis of the 69 candidate genes revealed five significantly over-represented pathways. (**A**) Bar chart of the top five terms: detection of chemical stimulus involved in sensory perception of smell (GO:0050911), digestion (GO:0007586), immunoregulatory interactions between a lymphoid and a non-lymphoid cell (R-HSA-198933), influenza A response (hsa05164), and inorganic ion transmembrane transport (GO:0098660). (**B**) Metascape enrichment network illustrating inter-term connectivity; node size corresponds to gene count and edge thickness reflects the number of shared genes. (**C**) Heatmap of Enrichr pathway associations for representative high-impact genes (rows) across the top five enriched pathways, with color intensity indicating −log_10_(*p*-value). Together, these panels highlight the convergence of sensory detection, metabolic processing, growth-factor and stress-response signaling, immune modulation and ion-transport modules in pharmacoresistant GGE.

Metascape [25] enrichment of the 69 shared high-impact genes revealed five significantly over-represented functional clusters (Figure 2A,B). The most enriched cluster, sensory perception of chemical stimulus, comprised seven olfactory and gustatory receptor genes (for example, OR10D3, OR8U1), suggesting a possible link to neurochemical processes. A digestion-related cluster, including MUC6, PNLIPRP2 and VDR, may reflect involvement of gastrointestinal or metabolic pathways [28,29]. Two immune-related clusters emerged: immunoregulatory interactions between lymphoid and non-lymphoid cells (LILRA2, HLA-DPB1, SIGLEC12, among others) and an influenza A response module (OAS2, SLC25A5), consistent with potential involvement of immune-related processes [30]. Finally, an inorganic ion transmembrane transport cluster (KCNJ12, P2RX5, RHBG, etc.) is consistent with prior associations between ion transport and epilepsy, including large-scale studies that have identified ion channelopathies as a major genetic risk factor in generalized epilepsies [30].

Enrichr pathway analysis [26,27] showed significant over-representation of Vitamin D metabolism and signaling (*p* = 2.73 × 10^−2^), FGF signaling (*p* = 4.59 × 10^−2^) and EGF receptor signaling (*p* = 5.45 × 10^−2^) among the 69 shared high-impact genes (Figure 2C). The mapping of VDR to the Vitamin D pathway is consistent with prior work linking vitamin D signaling to neuronal function and immune regulation, while MAP2K3 and RASA4 are components of MAPK- and Ras-related signaling pathways. Further enrichment of the insulin/IGF–MAPK cascade and oxidative stress response may be consistent with roles for growth-factor signaling and redox processes. Trends toward integrin and Toll-receptor pathways hint at altered cell–matrix adhesion and innate-immune activation. Together, these results highlight pathways that may warrant further study in the context of pharmacoresistant GGE. These pathway-level findings should be interpreted as preliminary signals in a small cohort and require validation in larger studies.

## 4. Discussion

### 4.1. Genetic Landscape of Adult Pharmacoresistant GGE

To our knowledge, this is the first WGS study focused exclusively on adults with pharmacoresistant GGE. Prior large-scale efforts encompassed both pediatric and adult cases but did not isolate pharmacoresistant adult GGE [22]. By requiring variants to be “common” (present in ≥80% of participants) and predicted deleterious, either annotated as high-impact by snpEff (frameshift, nonsense, canonical splice-site) or predicted by the ensemble radial SVM or LR., we distilled the variant set to 133 unique variants (Table S4) across 69 genes (Table S1). This coding-centric approach enabled recovery of both established epilepsy genes and novel candidate loci, expanding beyond classic protein-truncating alleles identified in earlier exome sequencing studies of epileptic encephalopathies [31].

### 4.2. Implications for Pharmacoresistance

Our analysis revealed enrichment of genes involved in gastrointestinal and metabolic functions, suggesting that these findings may be consistent with differences in ASM absorption or metabolism. High-impact variants in MUC6, encoding a key intestinal mucin, could be relevant to mucosal or metabolic processes that affect drug handling [32]. Similarly, PNLIPRP2 variants may disrupt pancreatic lipase-mediated lipid digestion, which may be relevant to processes influencing the handling of lipophilic ASMs [33,34]. Recurring VDR (vitamin D receptor) mutations implicate a hormonal regulatory axis: vitamin D status modulates expression of cytochrome P450 enzymes critical for ASM metabolism, and deficiency correlates with increased seizure frequency in refractory cohorts [28]. Moreover, high-impact variants in chemosensory receptor genes, such as OR10D3, OR8U1, and TAS2R19, may be relevant to blood–brain barrier or signaling processes, although this remains to be determined [35].

### 4.3. Overlap with Established Epilepsy Genes

Four of our prioritized genes, APOL4, KMT2C, SON, and VDR, are represented on the LabCorp clinical epilepsy panel (Table S10), underscoring the possible translational relevance of our WGS findings. APOL4 is a member of the apolipoprotein L family, lipid-binding proteins associated with HDL particles [36], and studies have identified APOL4 as an immune correlate in the brain tumor microenvironment, implicating it in neuroinflammatory processes [37]. KMT2C pathogenic loss-of-function variants underlie Kleefstra syndrome-2, a disorder in which seizures affect most patients [38]. SON haploinsufficiency causes Zhu-Tokita-Takenouchi-Kim (ZTTK) syndrome, characterized by global developmental delay and seizures in over 90% of reported cases [39]. Finally, VDR polymorphisms have been linked to epilepsy susceptibility, with the BsmI/ApaI/TaqI haplotype conferring increased risk for temporal lobe epilepsy [29]. Recovery of these four panel genes supports our variant-prioritization pipeline and endorses the integration of comprehensive WGS into clinical workflows for adult pharmacoresistant GGE. Furthermore, the relationship of these four genes and the pharmacoresistant GGE may be useful to investigate further in future studies.

### 4.4. Additional Epilepsy-Associated Coding Variants

In addition to the four panel-overlapping genes, we identified additional loci with prior links to epileptic phenotypes among the high-confidence variants shared by ≥80% of participants (Tables S1 and S4). Selected examples include HTR3D, which encodes a 5-HT_3_ serotonin receptor subunit whose dysfunction produces proconvulsive effects in rodent models [40]; LMLN2, which participates in processing synaptic glycoproteins such as reelin and LGI1, mutations of which cause lateral temporal lobe epilepsy [41]; PABPC3, a poly(A)-binding protein essential for mRNA translation, with de novo variants reported in rare epileptic encephalopathies [42]; RASA4, a calcium-dependent Ras GTPase-activating protein implicated in cortical dysplasia-related epilepsy [43]; and SERPINI1 (neuroserpin), in which pathogenic variants cause progressive myoclonus epilepsy [44].

Several prioritized variants were significantly enriched in the pharmacoresistant GGE cohort compared with individuals with GGE from the Epi25 reference dataset [22], suggesting a possible association between these variants or the genes they affect and the pharmacoresistant characteristics of our GGE cohort (Table S11). Variant-level metrics for all exonic variants, including allele counts and deleteriousness predictions across multiple tools, are provided in Supplementary Table S12.

### 4.5. Neuroinflammation and Signaling Pathways

Immune-related pathways were prominently featured among our shared high-impact variants. LILRA2, an activating leukocyte immunoglobulin-like receptor that senses cleaved immunoglobulins [45]; SIGLEC12, a sialic acid-binding lectin involved in both innate and adaptive immune regulation [46]; and OAS2, an interferon-induced 2′-5′ oligoadenylate synthetase critical for antiviral defense [47], all carried predicted loss-of-function or deleterious alleles [30]. These findings support a possible role for neuroinflammation in seizure persistence, consistent with evidence that cytokine-mediated synaptic modulation can lower seizure thresholds.

High-impact variants clustered in ion transporter genes, most notably KCNJ12, whose dysfunction is linked to epileptic phenotypes; P2RX5, a purinergic receptor that modulates neuronal excitability; and RHBG, an ammonium transporter essential for pH balance. This enrichment underscores the role of ionic homeostasis in maintaining neuronal network stability. Disruption of these channels is a well-established mechanism in generalized epilepsies [22], suggesting that such channelopathies may synergize with neuroinflammatory processes to drive hyperexcitability in pharmacoresistant GGE.

The identification of rare or syndromic genes in individuals with GGE supports the notion that GGE exists on a phenotypic spectrum, with seizures representing the most prominent or earliest clinical manifestation of a broader, often subclinical disorder. The convergence of multiple genetic findings in our cohort highlights shared biological pathways, reinforcing the concept of GGE as a network disorder. Disruption of key functional hubs within this network may not only predispose individuals to seizures but also contribute to pharmacoresistance, underscoring the importance of pathway-level insights for advancing precision treatment strategies.

In addition to immune signaling, oxidative stress and redox regulation have been implicated in epileptogenesis. Reactive oxygen species generated through NADPH oxidase (NOX), particularly NOX2, have been associated with neuronal hyperexcitability, while antioxidant pathways such as Nrf2 and thioredoxin contribute to the maintenance of redox balance. Experimental studies suggest that modulation of these pathways, including NOX inhibition and activation of Nrf2-dependent responses, can reduce seizure susceptibility and may have antiepileptogenic effects [48]. In this context, the enrichment of oxidative stress–related pathways in our analysis may be relevant, although further work is needed to determine their role in pharmacoresistant GGE.

### 4.6. Limitations and Future Directions

While this pilot analysis in ten phenotyped adults establishes a foundational framework for genomic discovery in pharmacoresistant GGE, expanding the size, ancestral diversity, and GGE status of the cohort will improve variant detection and strengthen generalizability. Although 18 eligible patients were invited and 10 participated, the small sample may still be subject to selection bias and may not fully represent the broader population of adults with pharmacoresistant GGE. Due to the small sample size, we emphasize that comparisons of variant frequencies in the refractory GGE cohort with reference populations are presented for exploratory and descriptive purposes; sampling variance may have led to spurious results. The sensitivity of variant prioritization to the chosen frequency threshold is shown in Supplementary Table S13. Although biogeographical ancestry was inferred and no significant imbalance relative to reference datasets was detected, the modest cohort size limited power for formal ancestry-stratified genetic modeling. Although we used variant frequencies from publicly available datasets as references, the study would be strengthened by the inclusion of an internal comparison group, particularly adults with drug-responsive GGE. Without such a group, it is not possible to determine whether the prioritized variants are specific to pharmacoresistance or reflect GGE more broadly. While the reference datasets provide useful population-level context, they are not matched to our cohort with respect to diagnosis, sequencing platform, or ancestry. Accordingly, variant-level and pathway-level findings in this cohort should be interpreted as hypothesis-generating rather than evidence of pharmacoresistance-specific genetic risk. Future studies incorporating a prospectively recruited, ancestry-matched pharmacoresponsive GGE cohort analyzed using the same sequencing and analytical framework would allow clearer identification of variants associated with pharmacoresistance.

Additionally, while short-read whole-genome sequencing was sufficient for identifying germline variants in this study, more comprehensive profiling of adults with refractory GGE would benefit from long-read sequencing to better characterize structural variants and copy number variation. Complementary functional validation will be useful to confirm mechanistic roles for prioritized variants. Future investigations that integrate larger, multiethnic populations with transcriptomic and epigenomic profiling will illuminate regulatory effects and gene-environment interactions. In parallel, longitudinal pharmacokinetic studies can directly link genotype with ASM exposure. Ultimately, prospective trials that stratify antiseizure medication selection by genetic profile will test the clinical impact of genomics-guided therapy in refractory adult GGE.

## 5. Conclusions

This pilot WGS investigation of coding variants in adult pharmacoresistant GGE identified variants in established panel genes along with novel high-impact variants in loci that implicate drug absorption, metabolic regulation, neuroimmune interactions, and ion transport. The recovery of four genes represented on a commercial epilepsy panel supports future prioritization of these genes. The identification of additional candidate pathways and a handful of variants observed in our pharmacoresistant GGE cohort at a frequency significantly higher than in a large reference GGE cohort provides a foundation for future studies aimed at clarifying mechanisms of drug resistance in genetic generalized epilepsy.

## Figures and Tables

**Table 1:** Patient demographics, clinical characteristics, and treatment histories.

Patient Number	Age (Years)	Gender	Race	Age of Epilepsy Diagnosis (Years)	Family History of Epilepsy	Seizure Type	History of Generalized Status Epilepticus	Past ASMs	Current ASMs
1	29	M	black	14	No	GTC, AB	Yes	VPA, PHT	LMG, ZNS, LEV, PRP, CLB
2	36	M	white	17	FDR	GTC, AB	No	VPA, LEV, LCM	ZNS, BRV
3	34	F	white	31	SDR	GTC, AB	Yes	VPA, LEV, LMG, OXC	ZNS, BRV, CLN
4	20	M	other-ind sub	12	No	GTC	No	LEV	ZNS, BRV, CLB
5	23	F	other-ind sub	13	No	GTC, M	No	LEV	VPA, TPM, LMG
6	49	F	other-mid es	19	No	GTC, M	No	LEV, PHT, LCM	VPA, ZNS, PRP
7	38	F	black	4	FDR	GTC, AB	Yes	LMG, TPM, OXC, CBZ, ECL, PHB	VPA, LEV, PHT
8	52	F	white	12	No	GTC, AB	No	CBZ, LMG, LEV, PHB, PRP	VPA, BRV
9	31	F	white	10	No	GTC, AB, M	No	LEV, PHT, TPM	LMG, ZNS
10	30	F	hispanic	5	SDR	GTC	Yes	LMG, CBZ	VPA, PRP, BRV, CBT

Abbreviations: M, male; F, female; FDR, first-degree biological relative (parent, sibling, offspring); SDR, other than FDR; GTC, generalized tonic–clonic seizure; AB, absence seizure; M, myoclonic seizure; ASM, antiseizure medication; BRV, brivaracetam; CBT, Cenobamate; CBZ, Carbamazepine; CLB, clobazam; CLN, clonazepam; ECL, eslicarbazepine; LCM, lacosamide; LEV, levetiracetam; LMG, lamotrigine; OXC, oxcarbazepine; PHB, phenobarbital; PHT, phenytoin; PRP, perampanel; TPM, topiramate; VPA, valproate sodium; ZNS, zonisamide.

## Data Availability

The sequencing data generated in this study have been deposited in the NCBI Sequence Read Archive (SRA) under BioProject accession PRJNA1283224. The BioProject and associated SRA metadata are available at https://dataview.ncbi.nlm.nih.gov/object/PRJNA1283224?reviewer=2vv725voeckms8167924dq1rit in read-only format (accessed on 10 May 2026).

## Supplementary Materials

The following supporting information can be downloaded at: https://www.mdpi.com/article/10.3390/brainsci16050521/s1, Table S1: Genes affected by shared high-impact variants present in ≥80 percent of adults with pharmacoresistant genetic generalized epilepsy; Table S2: Germline variant filtering summary (SNPs and Indels); Table S3: Commonality of germline variants among 10 samples; Table S4: Shared high-impact variants present in ≥80 percent of adults with pharmacoresistant genetic generalized epilepsy; Table S5: Genomic distribution and functional classification of variants identified in patients with intractable genetic generalized epilepsy; Table S6: Deleteriousness of SNVs; Table S7: Comparison of the distributions of ancestry estimates in our pharmacoresistant GGE cohort and the three reference groups; Table S8: Comparison of self-reported race with ancestry estimates in our pharmacoresistant GGE cohort.; Table S9: Distribution of variant type among the 133 variants of interest; Table S10: Genes included in the LabCorp Clinical Epilepsy Gene Panel; Table S11: Variants significantly enriched in pharmacoresistant genetic generalized epilepsy compared with the Epi25 genetic generalized epilepsy cohort; Table S12: Annotation and deleteriousness predictions for all exonic variants; Table S13: Number of exonic variants with either (i) LR ensemble model predicting deleterious, (ii) SVM ensemble model predicting deleterious, or (iii) snpEff predicting high-impact and which are found in at least N patients for N = 1,2,…,10.

## Abbreviations

The following abbreviations are used in this manuscript:

ASM	Anti-seizure medication
BRV	Brivaracetam
CBT	Cenobamate
CBZ	Carbamazepine
CLB	Clobazam
CLN	Clonazepam
CNS	Central nervous system
DNA	Deoxyribonucleic acid
EGF	Epidermal growth factor
FGF	Fibroblast growth factor
GATK	Genome Analysis Toolkit
GGE	Genetic generalized epilepsy
gDNA	Genomic DNA
gnomAD	Genome Aggregation Database
GO	Gene Ontology
GTC	Generalized tonic–clonic seizure
GWAS	Genome-wide association study
HDL	High-density lipoprotein
IGE	Idiopathic generalized epilepsy
IRB	Institutional Review Board
LEV	Levetiracetam
LGI1	Leucine-rich glioma-inactivated 1
LMG	Lamotrigine
LoF	Loss of function
MAPK	Mitogen-activated protein kinase
PBMC	Peripheral blood mononuclear cell
PCR	Polymerase chain reaction
PHB	Phenobarbital
PHT	Phenytoin
PRP	Perampanel
QC	Quality control
RCF	Relative centrifugal force
SIFT	Sorting intolerant from tolerant
SNP	Single nucleotide polymorphism
SNV	Single nucleotide variant
snpEff	SNP effect predictor
SRA	Sequence Read Archive
SUDEP	Sudden unexpected death in epilepsy
UTR	Untranslated region
VCF	Variant Call Format
VDR	Vitamin D receptor
VPA	Valproate sodium
WGS	Whole-genome sequencing
ZNS	Zonisamide
